# Supplementary material for: Evaluating spoken language as a biomarker for automated screening of cognitive impairment
Source: Commun Med (Lond). 2025 Dec 12;6:6. doi: 10.1038/s43856-025-01263-1 (PMC12770543; doi:10.1038/s43856-025-01263-1)
Supplement: Supplementary file 2 — Supplementary Materials [file 43856_2025_1263_MOESM2_ESM.pdf]

Supplementary Materials

DementiaBank Data

Our predictive modelling approach uses spontaneous speech recordings from two DementiaBank datasets for training and testing. We used the Alzheimer's Dementia Recognition through Spontaneous Speech only (ADReSSo) dataset from DementiaBank with speech recordings (N=237), acoustically pre-processed and balanced in terms of age and gender, as described in Table 1. The ADReSSo challenge has been proposed for systematic comparison of machine learning (ML) approaches for Alzheimer's disease (AD) detection and severity prediction using spontaneous speech data from the *Cookie Theft* picture description task from the Boston Diagnostic Aphasia Examination (BDAE).[1] The challenge baseline[2] achieved an accuracy of 64.8% and 77.5% on the test set using a Support Vector Machine (SVM) classifier on acoustic and linguistic features, respectively.

Additionally, we used the Lu corpus from DementiaBank set,[3] never seen during model training and used only for a final test (referred to as *external test*). Both datasets include picture descriptions produced by participants experiencing normal ageing (cognitively normal (CN)) and participants with an Alzheimer's disease and related dementias (ADRD) diagnosis.

Supplementary Table 1: DementiaBank datasets. Characteristics of participants in ADReSSo training and test datasets, as well as the Lu corpus. Note that the latter does not provide individual MMSE scores.

	ADReSSo train (N=166)		*ADReSSo* test (N=71)		*Lu* test (N=54)	
Cognitive Group	AD	CN	AD	CN	ADRD	CN
Participants	87	79	36	35	27	27
Sex (% male)	33%	34%	39%	37%	48%	33%
Age	69.7 (6.8)	66 (6.3)	68.5 (7.1)	66.1 (6.5)	79 (9)	79.7 (10.6)
MMSE	17.4 (5.3)	29 (1.2)	18.9 (5.8)	28.9 (1.3)	–	–

Machine Learning Pipeline

Supplementary Fig. 1 illustrates the proposed ML pipeline for ADRD detection and severity predictions. We explored acoustic, linguistic and six multimodal feature combinations using early fusion methods.[4]

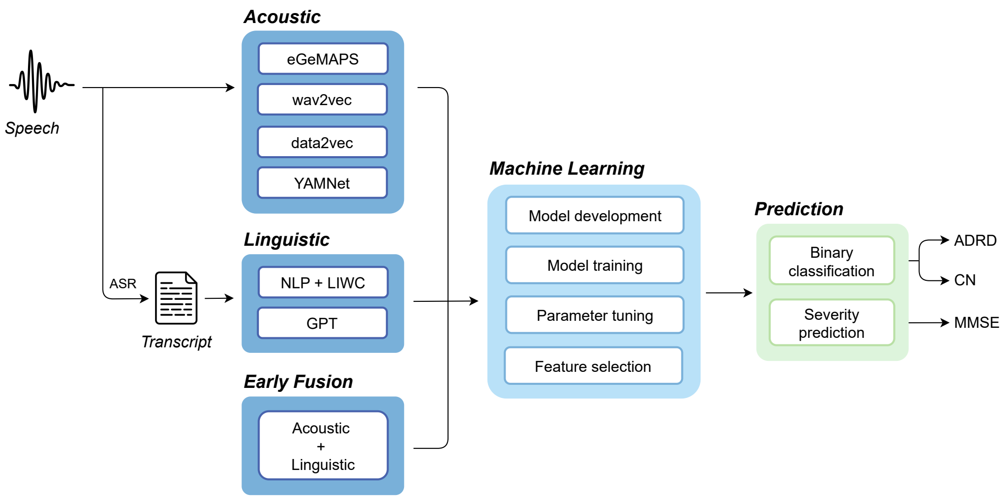

Supplementary Fig. 1: Proposed ML pipeline for cognitive health assessment. Analysis used for screening of cognitive health and MMSE prediction from spoken language.

Acoustic and Linguistic Features

We extracted acoustic and linguistic features using both conventional and pre-trained deep learning models (see Supplementary Fig. 2). We also analysed three multimodal feature set combinations using early fusion methods. We selected OpenAI's Whisper (*medium.en* model, version 20240930) as the automatic speech recognition (ASR) system to further extract linguistic features from transcribed text. One transcript was obtained from each audio file, i.e., one per participant.

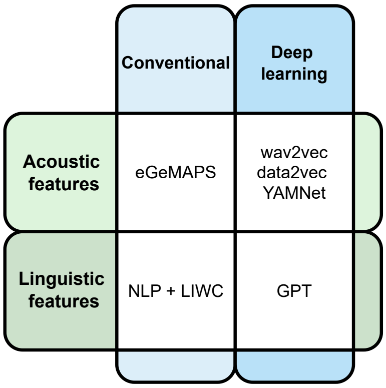

Supplementary Fig. 2: Feature extraction from speech and language. We extracted acoustic and linguistic features using both conventional and pre-trained deep learning models.

Acoustic features: We used the extended Geneva Minimalistic Acoustic Parameter Set (eGeMAPS) extracted directly from open-source Speech and Music Interpretation by Large-space Extraction (OpenSMILE)[5] with proven usefulness for paralinguistic acoustic feature extraction.[6] It consists of 88 features per speech sample, including frequency, spectral, and energy- related parameters that capture various aspects of voice quality, prosody, and speech dynamics.

Additionally, we used three deep neural embeddings designed to extract feature representations from audio data: wav2vec[7] using a vector size of 768, data2vec[8] using a vector size of 768, and YAMNet, which predicts audio events from 521 classes, using a vector size of 1024.

Linguistic features: We used OpenAI's GPT embeddings (*text-embedding-3-small* model) to represent participants' transcripts, with a vector size of 1536. Using conventional approaches based on domain knowledge, we extracted 100 NLP-based features, including lexical, semantic, and psycholinguistic features, as described in Table 2. Analytical thinking is a factor-analytically derived metric intended to capture logical, formal, and hierarchical thinking. It is based on the relative use of function words and computed by weighting function word frequencies by their factor loadings, placing each text on a narrative-to-analytical continuum.[9,10] Linguistic variables as highlighted in the SHAP plot (Fig. 3) and defined in LIWC-22 Psychometrics Manual,[11] is a normalised feature that aggregates the frequency of various linguistic dimensions, including function words, determiners, prepositions, auxiliary and common verbs, adverbs, conjunctions, negations, adjectives and quantities. These sub-features are also considered individually in the analysis.

Supplementary Table 2: NLP-based linguistic features extracted. Description of the lexical, semantic and psycholinguistics features extracted for predictive modelling.

Features	Description
Lexical diversity and semantic complexity (N=5)	
Type-Token Ratio (TTR)	The ratio of unique words (types) to total words (tokens) in a transcript, adjusted for text length. Lower TTR indicates less diverse vocabulary usage and less lexical richness
Propositional Idea Density (PID)	The number of expressed propositions – distinct facts or notions contained in a text with verbs, adjectives, adverbs, prepositions, and conjunctions – divided by the total number of words. These are considered as part-of-speech (POS) tags derived from NLTK Python toolkit. Lower PID suggests simpler language, while higher PID reflects a greater number of ideas in a concise form.
Brunet's index	A measure of lexical richness based on the variation in word types (part-of-speech) relative to the total word count. Lower values indicate higher lexical richness.
Honore's index	A measure of lexical diversity focused on the frequency of hapax legomena (words that appear only once). Lower Honore's Index values reflect reduced lexical variety.
Consecutive duplicate words	The proportion of duplicated words/phrases with reference to the total number of words/phrases.
Lexical and semantic psycholinguistics (N=95)	
LIWC	Different categories are extracted using LIWC-22 Dictionary, including: word count, summary language variables (e.g., analytical thinking, clout, authenticity, and emotional tone), general descriptor categories (words per sentence, percent of target words captured by the dictionary, and percent of words in the text that are longer than six letters), standard linguistic dimensions (e.g., percentage of words in the text that are pronouns, articles, adverbs, verbs), word categories tapping psychological constructs (e.g., affect, cognition, biological processes, drives) personal concern categories (e.g., home, leisure activities), informal language markers (assents, fillers)

Machine Learning Models

After data pre-processing, we evaluated various ML models on their performance at predicting (1) positive or negative ADRD and (2) individual Mini-Mental State Examination (MMSE) scores. For the ADRD binary classification task, we evaluated the following models:

- Logistic Regression (LR): L1 (Lasso) or L2 (Ridge) regularisation, with a value in $[10^{-5}, 10^{2}]$, with the solver being either 'liblinear' (more efficient for small datasets) or 'saga' (supports both penalties), determined by hyperparameter optimisation.
- SVM: Regularisation between $[10^{-4}, 10^{3}]$, gamma values from 'scale', 'auto', or random values in $[10^{-6}, 1]$, with 'linear' or 'rbf' kernels, determined by hyperparameter optimisation.
- Random Forest (RF): Gini entropy, number of estimators between $[50, 500]$ and a max depth between $[3, 20]$ given the training data size of 166 samples, determined by hyper-parameter optimisation.
- Multilayer Perceptron (MLP): Initial learning rate sampled between $[0.001, 0.01]$, logistic activation function, batch sizes selected from $[16, 32, 64, 128, 166]$ (166 is the total number of recordings available for training), hidden layer size of 400; trained using stochastic gradient descent with an adaptive learning rate; L2 regularization α sampled between $[10^{-4}, 10^{-3}]$, determined by hyperparameter optimisation.
- Extreme Gradient Boosting Decision Tree (XGBoost): Learning rate between $[0.01, 0.5]$, number of estimators between $[50, 500]$, max depth between $[1, 10]$, subsample ratio between $[0.01, 0.99]$, L1 regularisation α between [0, 0.001], determined by hyperparameter optimisation.

For the MMSE prediction regression task, we evaluated the following models:

- Ridge Regression (RR): L2 regularisation α between $[10^{-3}, 10]$, determined by hyperparameter optimisation.
- Support Vector Regression (SVR): Regularisation C in $[10^{-2}, 10^{2}]$, gamma values from 'scale' or 'auto', with 'linear' or 'rbf' kernels, determined by hyperparameter optimisation.

- Random Forest Regressor (RFR): Number of estimators between $[50, 200]$, max depth between $[5, 10]$, minimum samples split in $[2, 5]$, minimum samples per leaf between $[1, 2]$, determined by hyperparameter optimisation.
- MLP Regressor: Initial learning rate sampled between $[10^{-3}, 10^{-1}]$, batch sizes selected from $[16, 32]$, hidden layer size set by hyperparameter optimisation; trained with stochastic gradient descent, L2 regularisation α in $[10^{-3}, 10^{-2}]$.
- XGBoost Regressor: Learning rate between $[0.01, 0.3]$, number of estimators between $[50, 200]$, max depth between $[2, 6]$, subsample ratio fixed at 0.5, column sample ratio fixed at 0.5, L1 regularisation α in $[0, 1]$, and gamma between $[0, 0.4]$, determined by hyperparameter optimisation.

All hyperparameter optimisation was conducted using a 10-fold cross-validation strategy on the training set.

Evaluation Metrics

In this section, we discuss the evaluation metrics used to assess the performance of the proposed machine learning models in classification and regression tasks.

In classification, we used four evaluation metrics in our study, including specificity, sensitivity, receiver operating characteristic area under the curve (ROC-AUC). Each metric provides important information about the performance of the model, and their combined use helps provide a comprehensive picture of the model's predictive ability. Understanding these metrics can help healthcare providers to assess the reliability and usefulness of these models in clinical practice.

- ROC-AUC: summarizes the model's performance across all classification thresholds by plotting the true positive rate against the false positive rate. We selected ROC-AUC as the primary evaluation metric as it is based on the predicted probability scores, providing a comprehensive assessment of the model's ability to distinguish true ADRD cases while minimizing false positives across all classification thresholds.
- Sensitivity (and equivalently, recall): the proportion of true positive predictions among all actual positive cases. It measures the model's ability to correctly identify individuals who are at risk of ADRD. A high sensitivity indicates that the model is effective at identifying true positive cases.

$$\text{Sensitivity} = \frac{\text{TP}}{\text{TP} + \text{FN}},$$

  where TP, TN, FP, and FN refer to True Positives, True Negatives, False Positives, and False Negatives, respectively.
- Specificity: measures the model's ability to correctly identify individuals who are not at risk of a ADRD. A high specificity indicates that the model is effective at identifying true negatives, i.e., those who are not at risk.

$$\text{Specificity} = \frac{\text{TN}}{\text{FP} + \text{TN}}$$

- Accuracy: The proportion of correct predictions – both true positives and true negatives – among all cases.

$$\text{Accuracy} = \frac{\text{TP} + \text{TN}}{\text{TP} + \text{TN} + \text{FP} + \text{FN}}$$

In MMSE prediction, we used two evaluation metrics:

- mean absolute error (MAE): measures the average magnitude of the errors in the predictions. It is the average absolute difference between the predicted and actual values. A lower MAE indicates better predictive performance.

$$\text{MAE} = \frac{1}{n}\sum_{i=1}^{n} |y_i - \hat{y}_i|,$$

  where y_i is the actual value, $\hat{y}_i$ is the predicted value, and n is the number of samples.
- Root Mean Square Error (RMSE): the square root of the average of the squared differences between the predicted and actual values. RMSE gives higher weight to larger errors, making it more sensitive to outliers than MAE. A lower RMSE indicates a better fit to the data.

$$\text{RMSE} = \sqrt{\frac{1}{n}\sum_{i=1}^{n}(y_i - \hat{y}_i)^2}$$

Model Calibration and Reliability

This section presents the results of the RF-NLP model's reliability and calibration analysis. Supplementary Fig. 3a shows the model is well-calibrated. There is some variability in the calibration across the bootstrap runs, but the general trend remains close to the diagonal, indicating that the model is fairly robust. The reliability analysis shows that the risk scores can be considered reliable when considering both positive and negative ADRD predictions together. This can be seen by observing the small gaps in the top plot of Supplementary Fig. 3b and the distance between the average accuracy and average confidence in the lower plot.

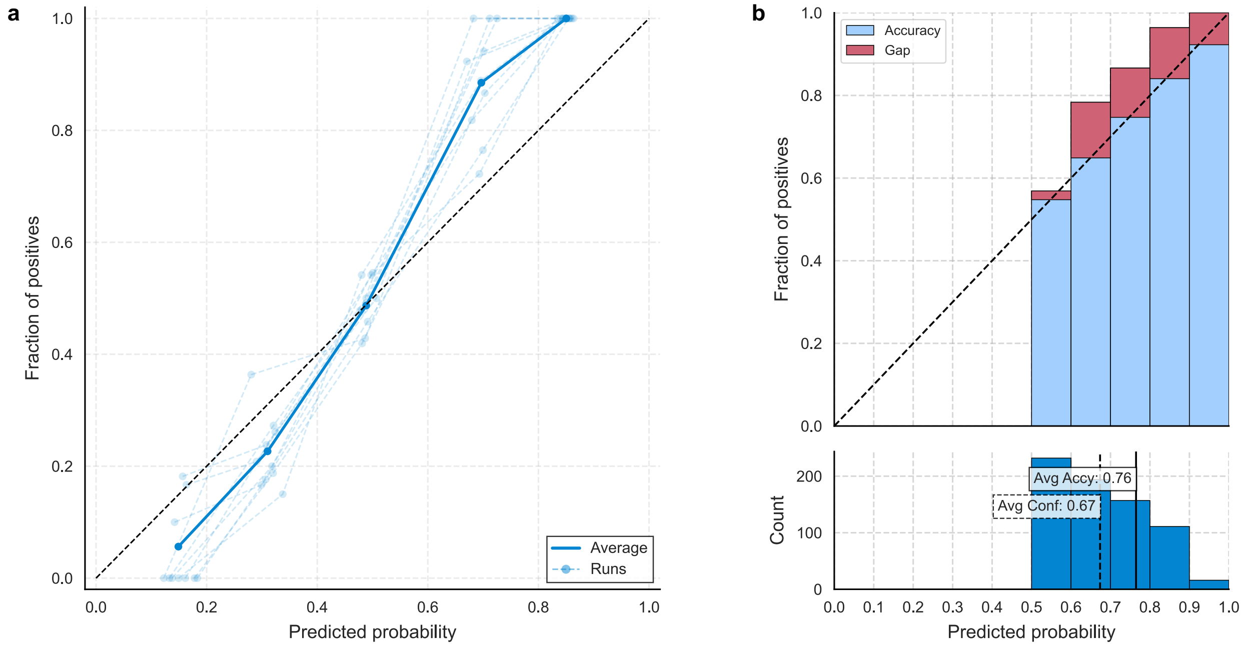

Supplementary Fig. 3: Model calibration and reliability plots. **a** The calibration plot shows the mean ADRD predicted risk against the proportion of positive cases for the best model, evaluated on the test set. The 10 bootstrap runs are represented in lighter colour. **b** The reliability plot: top shows the model confidence (for positive and negative ADRD cases) against accuracy on the test set. The gap represents the difference between average accuracy and confidence per bin, with an ideal gap of 0. Bottom shows a histogram of model confidence levels on the test set.

Classification Performance of All Models and Feature Sets Tested

We evaluated each classifier (detailed in Supplementary Section) on different acoustic and linguistic feature sets using conventional knowledge-based features as we as those extracted fusing deep learning (see details in Supplementary Section). Additionally, we analysed three multimodal (i.e., combining acoustic and linguistic) feature combinations using early fusion methods. Table 3 presents the results of all feature sets tested in binary classification with the best-performing model of each feature selected based on the highest ROC-AUC on the validation set.

Supplementary Table 3: Results of the feature sets tested for ADRD detection in binary classification. Evaluation metrics include sensitivity, specificity, ROC-AUC and accuracy, reported as mean (standard deviation)% for the 10-fold cross-validation (CV). The bold row shows the chosen model for our interpretable predictive modelling.

Feature	Model	Sensitivity	Specificity	ROC-AUC	Accuracy
Linguistic					
**NLP**	**RF**	**78.8 (16.7)**	**72.1 (13.4)**	**83.5 (8.9)**	**75.3 (9.4)**
GPT	MLP	79.3 (13.5)	76.2 (18.1)	87.5 (7.7)	77.7 (12.3)
Acoustic					
eGeMAPS	LR	69.3 (11.7)	70.5 (20.3)	73.1 (12.3)	69.8 (8.3)
wav2vec	LR	78.1 (9.8)	67.0 (16.3)	78.6 (11.1)	72.9 (10.2)
data2vec	MLP	75.3 (14.5)	73.0 (16.5)	81.9 (13.0)	74.4 (12.3)
YAMNet	SVM	72.8 (14.3)	69.6 (15.9)	77.5 (12.4)	71.1 (11.2)
Fusion					
eGeMAPS + NLP	LR	72.4 (10.6)	76.2 (18.1)	77.4 (10.4)	74.1 (9.1)
eGeMAPS + GPT	LR	76.0 (12.0)	86.2 (13.1)	87.1 (8.3)	80.7 (10.7)
data2vec + NLP	XGBoost	83.8 (7.9)	78.6 (11.1)	87.4 (10.3)	81.3 (6.9)

Performance on Demographic Groups

We evaluated model performance on different demographic groups based on sex (female, male), and age (50-59, 60-69, 70-80 years). To do this, we split the predictions made by the proposed model (with thresholds of $> 50\%$ = Positive and $< 50\%$ = Negative) on the test set by demographic group and calculate the mean accuracy and standard deviation for each group. These results, along with the number of participants in each demographic group and binary label proportions, are shown in Supplementary Table 4.

The likelihood of a positive prediction across demographics does not show high variations, even with an imbalance in the number of participants of the younger group. Furthermore, Supplementary Table 4 shows that participants in the oldest age group (70-80 years) have a higher likelihood of a positive ADRD prediction than the younger groups. Overall, this analysis suggests our model is a fair classifier, demonstrating high demographic parity.[12]

Supplementary Table 4: Model performance on demographic group splits. Mean (95% CI) % accuracy of the RF-NLP model for the female/male and age groups on the test set with 10 bootstrap repeats. We also show the likelihood of a positive ADRD prediction, $P(\hat{y} = 1)$, for each demographic and the proportion of positive and negative labels on the test set, Pos : Neg.

Split	Total	Accuracy	$P(\hat{y}= 1 \mid \text{Demographic})$	Pos : Neg
Female	44	76.4 (74.6–78.1)	42.3 (38.6–46.0)	1 : 1.1
Male	27	76.7 (72.2–81.2)	43.3 (37.9–48.8)	1 : 0.9
50–59 years	14	83.6 (78.2–89.0)	42.1 (34.7–49.5)	1 : 1.3
60–69 years	27	75.9 (72.6–79.3)	37.4 (31.5–43.3)	1 : 1.2
70–80 years	30	73.7 (71.0–76.3)	47.7 (44.7–50.7)	1 : 0.8

Performance on MMSE Groups

We evaluated model performance on different cognitive groups based on participants' MMSE scores (see Table 5). We found that the higher the cognitive impairment (lower MMSE score) the higher the likelihood of a positive ADRD prediction of the RF model trained on NLP-based linguistic features. This finding supports Fig. 1, which shows that the model is more confident (i.e., makes less mistakes) for the moderate and severe cognitive impairment groups compared to the mild and CN. Additionally, Supplementary Fig. 4 shows that the number of true positives (TP) increases for participants with moderate cognitive impairment compared to mild, while the number of true negatives (TN) is the highest for the CN group.

Supplementary Table 5: Model performance on MMSE group splits. Mean (95% CI) % accuracy of the RF-NLP model for the MMSE groups on the test set with 10 bootstrap repeats. We also show the likelihood of a positive ADRD prediction for each group. Note that the test set included only two participants with MMSE scores in the severe group, which explains the metrics obtained.

Cognitive Group	Total	Accuracy	$P(\hat{y}=1 \mid \text{MMSE})$
CN	36	81.9 (76.8–87.1)	20.8 (15.0–26.6)
Mild	11	48.2 (43.8–52.6)	39.1 (34.7–43.5)
Moderate	21	78.6 (73.7–83.5)	78.6 (73.7–83.5)
Severe	2	100	100

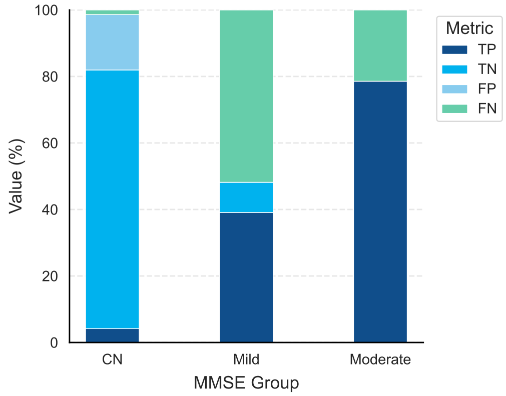

Supplementary Fig. 4: Distribution of model predictions for MMSE groups. TP, TN, FP, and FN refer to True Positives, True Negatives, False Positives, and False Negatives, respectively.

Risk Breakdown of Individual Predictions

Supplementary Fig. 5 shows four different predictions, each broken down by SHAP score contributions from individual features. This visualization can enable clinicians to explore the factors behind each model prediction. For example, Supplementary Fig. 5c shows a correct negative prediction (i.e., participant belongs to the CN group) with a predicted risk of 12%. Significant contributors to this prediction include frequent references to *family* and *lifestyle*, higher levels of *analytical thinking*, and less frequent use of *pronouns* and other linguistic variables.

Participant Word Transcription Proportion

We calculated Spearman's rank correlation using the participant word transcription proportion and individual MMSE scores. The analysis revealed a moderate positive correlation ($r = 0.53$, $p < .05$), meaning participants with higher MMSE had a higher proportion of participant speech in their transcriptions. Notably, participants in the moderate and severe cognitive groups, with lower mean participant transcription word proportion of 73.5% and 40.6%, respectively, received more frequent intervention from the administrator (e.g., "What else is going on?", "Can you tell me what else is going on in that picture?") (shown in Supplementary Table 6). This increased administrator input introduced noise into the linguistic features used by the ML model for predictions, which could explain the higher MAE obtained in MMSE prediction for the severe group.

Supplementary Table 6: Participant word transcription proportion by cognitive group. Results are reported as mean (standard deviation)% for transcripts analysed on the test set.

	CN	Mild	Moderate	Severe
Word proportion (%)	91.1 (15.2)	84.7 (13.8)	73.5 (17.6)	40.6 (29.4)

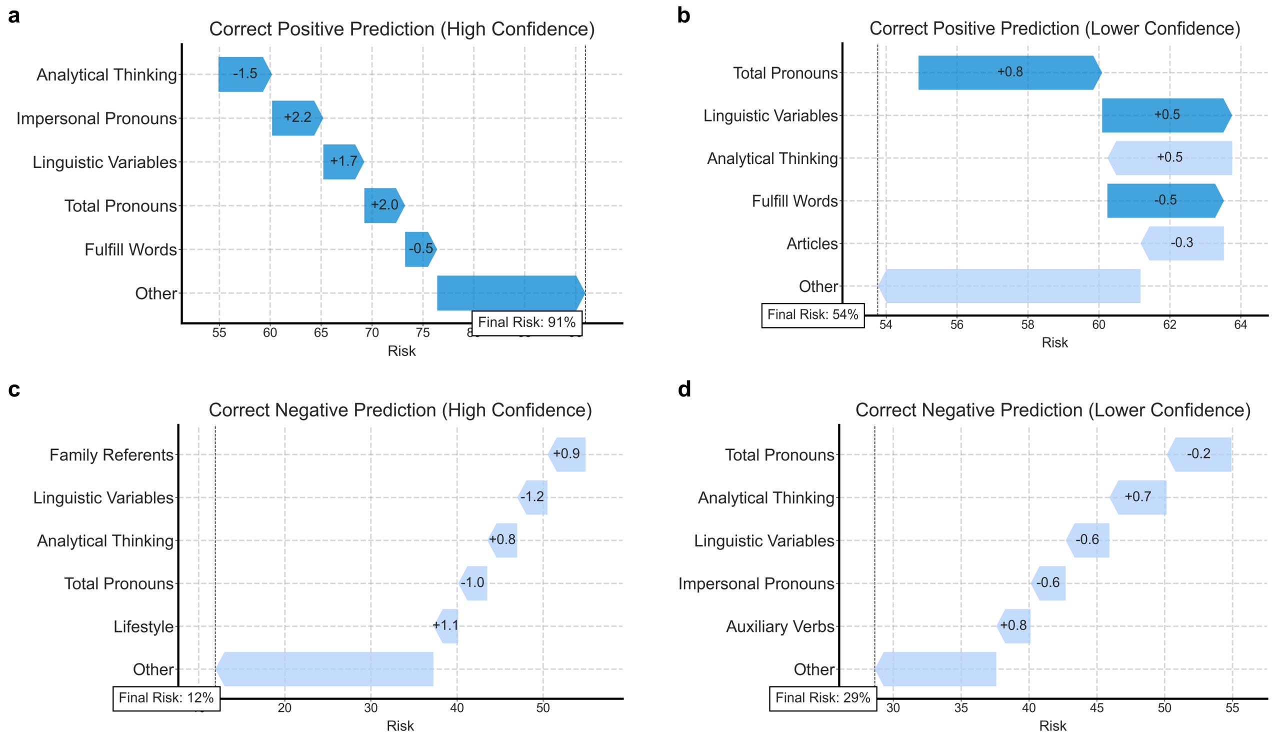

Supplementary Fig. 5: Feature importance results for individual predictions. This figure shows how each linguistic feature contributed to individual predictions based on SHapley Additive exPlanations (SHAP) values: a) a correct positive prediction (i.e., risk of ADRD) with a high risk score of 91% (participant in the moderate group, MMSE=13); b) a correct positive prediction with a lower risk score of 54% (participant in the mild group, MMSE=25); c) a correct negative prediction (i.e., CN) with a risk score of 12% (participant in the CN group, MMSE=29); d) a correct negative prediction with lower confidence and a risk score of 29% (participant in the CN group, MMSE=27); Here, the values on the arrows represent the normalised feature value in standard deviations from the mean.

Regression Performance of All Models Tested with NLP Features

We evaluated five models to predict participants' MMSE scores from natural language processing (NLP)-based linguistic features. Supplementary Table 7 shows the results for the 10-fold CV. The best-performing model was RFR, with lower MAE and RMSE.

Supplementary Table 7: MMSE prediction results for the 10-fold CV. MAE and RMSE results of the five regression models evaluated, reported as mean (standard deviation) for the 10-fold CV. The bold row shows the chosen model for our interpretable predictive modelling.

Model	MAE	RMSE
RR	7.5 (1.3)	6.0 (1.2)
SVR	6.4 (1.2)	5.3 (0.9)
**RFR**	**5.9 (0.7)**	**4.8 (0.5)**
MLP	8.3 (1.8)	6.6 (1.7)
XGBoost	5.9 (0.8)	4.8 (0.5)

Automatic Speech Recognition Robustness Across Cognitive Groups

We analyzed a representative subset comprising 61% of the DementiaBank dataset (N=145), covering at least 50% of speech samples from each cognitive group. We computed word error rate (WER) using the metric from HuggingFace (https://huggingface.co/spaces/evaluate-metric/wer). There was an overall WER of 15.7% (SD = 19.8%). Future iterations of this work could explore fine-tuning Whisper on annotated pathological speech datasets.

We further looked into the different cognitive groups. As expected, WER increased with cognitive impairment severity. As seen in Fig. 6, the highest error rates were observed in the severe impairment group (mean = 31.4%, SD = 12.8), while the cognitively normal (CN) group showed the lowest error rates (mean = 8.9%, SD = 9.1). These trends reflect known patterns of reduced fluency and increased disfluencies in advanced stages of dementia, which pose greater challenges for ASR systems.

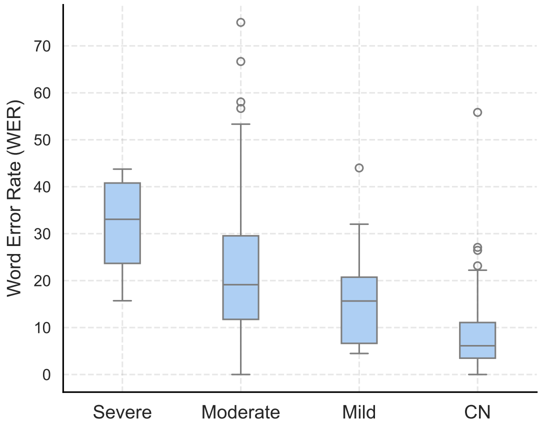

Supplementary Fig. 6: Word error rate by cognitive group. WER values were computed for a representative subset of the DementiaBank dataset (61%, N=145). Each boxplot shows the distribution of WER across cognitive groups based on MMSE scores. Higher WER values were observed in participants with more severe cognitive impairment, reflecting increased speech disfluencies and transcription difficulty.

Pilot Study: Multilingual Sensitivity Analysis

We conducted a sensitivity analysis by splitting the pilot data into English-only (N=14) and Spanish-only (N=8) subsets. We computed classification metrics for each subgroup using 10 bootstrap repeats and report 95% confidence intervals. Not surprisingly, the model performance drops for the Spanish-only subset (see Supplementary Table 8). While sensitivity remained comparable between English (71.0%, 95% CI: 61.8–80.2) and Spanish samples (67.5%, 95% CI: 52.8–82.2), specificity decreased in the Spanish group, leading to a drop in overall accuracy to 53.8% (95% CI: 47.7–59.8).

This drop likely reflects two factors: (1) lexical noise introduced by automated translation; (2) the higher average MMSE in the Spanish subgroup of 26.1 (3.6) vs. 24.2 (3.9) in the English subgroup, suggesting that many participants were near the cutoff (MMSE=26) where model uncertainty is higher, as demonstrated in Fig. 1. We also note differences in the distribution of cognitive impairment groups across subsets. The English subset included participants in the moderate group – where the model is more confident – whereas the Spanish subset only included individuals in the CN or MCI groups. We anticipate that performance on Spanish-only samples would improve with a larger cohort that includes individuals with more advanced cognitive impairment.

Supplementary Table 8: Multilingual sensitivity analysis. Classification performance metrics with 95% confidence intervals for English and Spanish subsets of the pilot dataset collected in-residence from older adults.

Subset	Sensitivity	Specificity	ROC-AUC	Accuracy
English	71.0 (61.8–80.2)	60.0 (42.7–77.3)	77.0 (70.8–83.2)	67.9 (61.8–73.9)
Spanish	67.5 (52.8–82.2)	40.0 (30.8–49.2)	45.6 (36.7–54.6)	53.8 (47.7–59.8)

References

[1] Goodglass H, Kaplan E, Weintraub S. BDAE: The Boston diagnostic aphasia examination. Lippincott Williams & Wilkins Philadelphia, PA; 2001.

[2] Luz S, Haider F, de la Fuente S, Fromm D, MacWhinney B. Detecting cognitive decline using speech only: The adresso challenge. arXiv preprint arXiv:210409356. 2021.

[3] Lanzi AM, Saylor AK, Fromm D, Liu H, MacWhinney B, Cohen ML. DementiaBank: Theoretical rationale, protocol, and illustrative analyses. American Journal of Speech-Language Pathology. 2023;32(2):426-38.

[4] Baltrušaitis T, Ahuja C, Morency LP. Multimodal machine learning: A survey and taxonomy. IEEE transactions on pattern analysis and machine intelligence. 2018;41(2):423-43.

[5] Eyben F, Wöllmer M, Schuller B. Opensmile: the munich versatile and fast open-source audio feature extractor. In: Proceedings of the 18th ACM international conference on Multimedia; 2010. p. 1459-62.

[6] Eyben F, Scherer KR, Schuller BW, Sundberg J, André E, Busso C, et al. The Geneva minimalistic acoustic parameter set (GeMAPS) for voice research and affective computing. IEEE transactions on affective computing. 2015;7(2):190-202.

[7] Baevski A, Zhou Y, Mohamed A, Auli M. wav2vec 2.0: A framework for self-supervised learning of speech representations. Advances in neural information processing systems. 2020;33:12449-60.

[8] Baevski A, Hsu WN, Xu Q, Babu A, Gu J, Auli M. Data2vec: A general framework for self-supervised learning in speech, vision and language. In: International Conference on Machine Learning. PMLR; 2022. p. 1298-312.

[9] Pennebaker JW, Chung CK, Frazee J, Lavergne GM, Beaver DI. When small words foretell academic success: The case of college admissions essays. PloS one. 2014;9(12):e115844.

[10] Jordan KN, Sterling J, Pennebaker JW, Boyd RL. Examining long-term trends in politics and culture through language of political leaders and cultural institutions. Proceedings of the National Academy of Sciences. 2019;116(9):3476-81.

[11] Boyd RL, Ashokkumar A, Seraj S, Pennebaker JW. The development and psychometric properties of LIWC-22. Austin, TX: University of Texas at Austin. 2022;10:1-47.

[12] Google. Measuring fairness;. Available from: `https://pair.withgoogle.com/explorables/measuring-fairness/`.
