# Supplementary material for: Evaluating spoken language as a biomarker for automated screening of cognitive impairment
Source: Commun Med (Lond). 2025 Dec 12;6:6. doi: 10.1038/s43856-025-01263-1 (PMC12770543; doi:10.1038/s43856-025-01263-1)
Supplement: Supplementary file 3 — Description of Additional Supplementary files [file 43856_2025_1263_MOESM3_ESM.pdf]

Description of Additional Supplementary Files

File name: Supplementary Data 1

Description: Source data for Fig. 1 and Fig. 2.

File name: Supplementary Data 2

Description: Source data for Fig. 3a and Fig. 3b.

File name: Supplementary Data 3

Description: Source data for Fig. 4.
